# Emerin deficiency does not exacerbate cardiomyopathy in a murine model of Emery–Dreifuss muscular dystrophy caused by an *LMNA* gene mutation

**DOI:** 10.1186/s12576-023-00886-0

**Published:** 2023-11-08

**Authors:** Eiji Wada, Kohei Matsumoto, Nao Susumu, Megumi Kato, Yukiko K. Hayashi

**Affiliations:** Department of Pathophysiology, Tokyo Medical University, Tokyo, Japan

**Keywords:** Emery–Dreifuss muscular dystrophy, Lamin a/c, Emerin, Cardiomyopathy, Fibrosis

## Abstract

Emery–Dreifuss muscular dystrophy (EDMD), caused by mutations in genes encoding nuclear envelope proteins, is clinically characterized by muscular dystrophy, early joint contracture, and life-threatening cardiac abnormalities. To elucidate the pathophysiological mechanisms underlying striated muscle involvement in EDMD, we previously established a murine model with mutations in *Emd* and *Lmna* (*Emd*^−/−^/*Lmna*^H222P/H222P^; EH), and reported exacerbated skeletal muscle phenotypes and no notable cardiac phenotypes at 12 weeks of age. We predicted that lack of emerin in *Lmna*^H222P/H222P^ mice causes an earlier onset and more pronounced cardiac dysfunction at later stages. In this study, cardiac abnormalities of EDMD mice were compared at 18 and 30 weeks of age. Contrary to our expectations, physiological and histological analyses indicated that emerin deficiency causes no prominent differences of cardiac involvement in *Lmna*^H222P/H222P^ mice. These results suggest that emerin does not contribute to cardiomyopathy progression in *Lmna*^H222P/H222P^ mice.

**Supplementary Information:**

The online version contains supplementary material available at 10.1186/s12576-023-00886-0.

## Introduction

Emery–Dreifuss muscular dystrophy (EDMD) is a rare genetic disorder characterized by progressive humeroperoneal muscle weakness, early-onset joint contractures, and cardiomyopathy with conduction defects [1]. Life-threatening progressive cardiomyopathy is prevalent in almost all EDMD patients [2, 3]. Among the several responsible genes identified for EDMD, the most common genetic subtypes are EDMD1 caused by emerin gene (*EMD*) mutations and EDMD2 caused by lamin A/C gene (*LMNA*) mutations. Emerin localizes predominantly to the inner nuclear membrane, and is ubiquitously expressed in most cells [4], but mutations in its gene cause tissue-specific phenotypes [5]. Lamin A/C is an intermediate filament protein that localizes underneath the nuclear membrane. Mutations in *LMNA* are known to cause a wide range of tissue-specific diseases, which are collectively referred to as the laminopathies [6]. Lamin A/C anchors nuclear envelope proteins, including emerin, to stabilize the nuclear architecture [7]. It is speculated that emerin and lamin A/C play roles in tissue-specific gene expression, cell signaling, and nuclear envelope integrity; however, the mechanisms as to how alterations in nuclear envelope proteins cause EDMD remain largely unknown.

In mouse models of EDMD, emerin-deficient *Emd*^*−/−*^ (Emd) mice do not show obvious dystrophic or cardiomyopathic phenotypes [8], and *Lmna*^H222P/H222P^ (H222P) knock-in mice recapitulate the cardiac phenotypes of EDMD patients, but only demonstrate limited phenotypes in skeletal muscle after the appearance of cardiac dysfunction [9]. Our recent research has provided evidence that emerin deficiency contributes to the worsening of muscular dystrophy in H222P mice from adolescence, whereas cardiac dysfunction is not prominent in both H222P and *Emd*^−/−^/*Lmna*^H222P/H222P^ double-mutant (EH) mice at 12 weeks of age [10]. Therefore, EH mice have provided valuable insights towards elucidating the pathological mechanism of skeletal muscle dystrophy in EDMD, which is independent from cardiac dysfunction.

To understand the tissue-specific roles of emerin in the background of the *LMNA* mutation, we investigated the progression of cardiac abnormalities in EDMD model mice in adulthood. We found that both H222P and EH mice showed prominent cardiomyopathy at 18 weeks of age, which was worse at the age of 30 weeks whereas cardiac involvement was not observed in Emd mice at these ages. In this report, we demonstrated that there was no pronounced difference in the progression of cardiac abnormalities in H222P and EH mice.

## Materials and methods

### Mice

Wild-type C57BL/6 J (WT), Emd, H222P, and EH mice were fed standard chow and water ad libitum. All mice were maintained in a specific pathogen-free facility with 12-h/12-h light/dark cycles. Life span of male and female H222P and EH mice was compared by a Kaplan–Meier survival curve. All animal experiments were approved by the Animal Care & Use Committee of Tokyo Medical University (Approval Number: R5-050) and were performed in accordance with the Guide for the Care and Use of Laboratory Animals published by the US National Institutes of Health.

### Echocardiography

Electrocardiograms from long axis M-mode images were recorded using the non-invasive ARIETTA prologue (Hitachi, Japan) with a 15.3-MHz transducer, as previously reported [10]. Briefly, mice were anesthetized with 3% isoflurane, and then sedated with continuous 1% isoflurane with their heart rate stabilized between 400 and 500 beats per minute during analysis.

### Histopathology

Isolated hearts were frozen in isopentane cooled in liquid nitrogen, and cut into transverse 10-μm thick sections using a Leica CM 3050S cryostat, and stained with hematoxylin and eosin (H&E), succinate dehydrogenase (SDH), and cytochrome c oxidase (COX). The intensity of SDH-stained or COX-stained area was normalized by the area of H&E stained to calculate the percentage of each enzyme in cardiac muscle from serial sections using NIH ImageJ software. For immunohistochemistry, 8-μm thick cryosections were fixed with 4% paraformaldehyde in PBS for 10 min at room temperature (RT). After blocking with 2% bovine serum albumin (BSA)/PBS, sections were incubated with primary antibodies at 37 °C for 1 h, and Alexa Fluor 488 or 568 secondary antibodies (1:1000; Thermo Scientific) with DAPI solution was used for detection. Alexa Fluor 568 phalloidin (A12380, Invitrogen) was used for staining actin filaments.

### Protein extraction and immunoblotting

Total proteins were isolated from cardiac sections with extraction RIPA buffer (Fujifilm) containing protease inhibitors and phosphatase inhibitors (Roche). Protein content was determined by BioPhotometer (Eppendorf), and the sample was mixed with 2 × sample buffer (Fujifilm). Protein extracts (30 µg) were analyzed by SDS-PAGE using gradient gels (5%–20% and 10%–20%) and transferred onto PVDF membranes by the semi-dry technique using the Trans-Blot Turbo system (Bio-Rad). The membranes were blocked with 1% BSA in Tris-buffered saline containing 0.05% Tween 20 for 1 h at RT, and then incubated with the primary antibody overnight at 4 °C. Membranes were then incubated with horseradish peroxidase-conjugated secondary antibodies (Thermo Scientific) for 1 h at RT. All bands were detected with Clarity Western ECL Substrate (Bio-Rad) using the ChemiDoc Imaging System (Bio-Rad). All data were analyzed as relative band intensities using Image Lab 5.0.

### Antibodies

Primary antibodies used were anti-periostin (NBP1-30,042, Novusbio), anti-β-catenin (06–734, Upstate), anti-PDGFRα (AF1062, R&D systems), anti-α-smooth muscle actin (SMA) (ab7817, Abcam), anti-β-actin (D6A8, Cell Signaling), anti-PGC1α (NBP1-04676, Novusbio), anti-NDUFAF1 (ab79826, Abcam), anti-ATP5A1 (ab14748, Abcam), anti-TFAM (ab131607, Abcam), anti-phospho mTOR (2971, Cell Signaling), anti-mTOR (2983, Cell Signaling), anti-phospho AKT (9271, Cell Signaling), anti-AKT (9272, Cell Signaling), anti-phospho Smad3 (2520, Cell Signaling), anti-Smad3 (2523, Cell Signaling), anti-phospho ERK1/2 (9101, Cell Signaling), and anti-ERK1/2 (9102, Cell Signaling). All antibodies were used at the dilutions recommended by the manufacturers.

### RNA isolation and quantitative reverse-transcription PCR (RT-qPCR)

Total RNA was extracted from mouse cardiac sections using RNeasy Plus Universal Mini kit (QIAGEN). The purity and integrity of the extracted RNA were determined using a NanoDrop One spectrophotometer (Invitrogen) according to the manufacturer’s instructions. Complementary DNA (cDNA) was synthesized using SuperScript VILO cDNA synthesis kit (Thermo Scientific) in accordance with the manufacturer’s instructions. Realtime RT-qPCR was performed using the QuantStudio 3 Real-Time PCR system using SYBR green master mix (Applied Biosystems). The primers used in this study are listed in Additional file 1: Table S1. All results were normalized using *Gapdh*, and gene expression levels were calculated by the ΔΔCT method of relative quantification. Data were expressed as the fold-increase versus the values of WT mice.

### Statistical analysis

Data are shown as the mean ± standard deviation (SD), and differences were determined by one-way ANOVA with the post-hoc Tukey multiple comparison test or the post-hoc Dunnett *t*-test. In cases where the means of two parameters were compared, differences were determined by the Welch’s t test. Statistical tests were two sided, and a *p*-value of less than 0.05 was considered to indicate a statistically significant difference between groups. All statistical analyses were performed using SPSS statistics 28 software (IBM).

## Results

### Life span

**Fig. 1:**
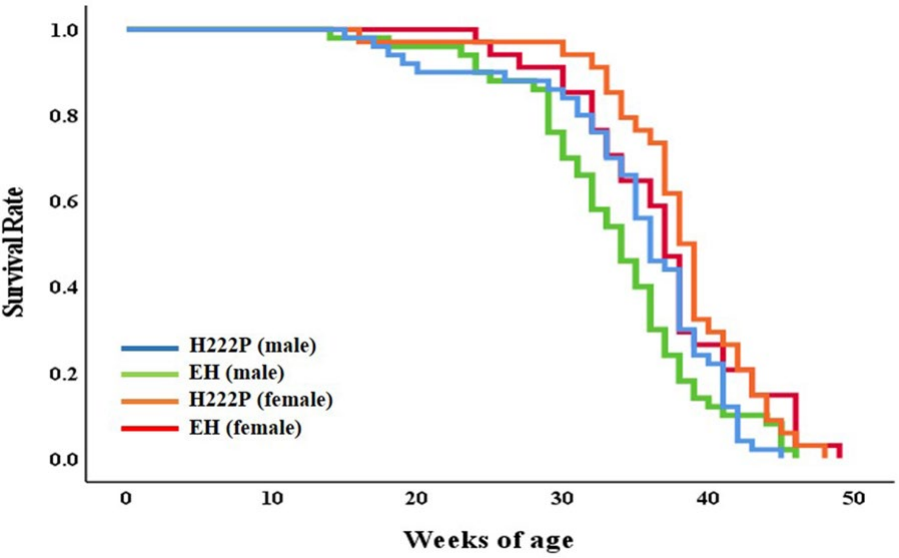
A Kaplan–Meier survival curve of male and female H222P and EH mice. No significant differences were observed in average life span (weeks of age) between H222P and EH mice, both in male and female mice

The three EDMD model mouse lines were indistinguishable with WT mice at birth, and had breeding productivity, as previously reported [8–10]. H222P mice had a slightly lower body weight in adulthood, and both H222P and EH mice had reduced body weights at 18 and 30 weeks of age, demonstrated a reduction in body weight with age [10]. Comparison of a Kaplan–Meier survival curve showed similar pattern among male and female H222P and EH mice (Fig. 1). The average life span of male H222P mice (34.8 ± 7.0 weeks, n = 50) and male EH mice (33.4 ± 6.6 weeks, *n* = 50) was not significantly different. Female mice had a slightly longer life span in both H222P (38.1 ± 5.7 weeks, *n* = 34) and EH mice (36.9 ± 6.1 weeks, *n* = 34), and there was no significant difference in the life spans of female H222P and EH mice. Male mice were used for further analyses of cardiomyopathy in EDMD model animals.

### Cardiac function and histology

**Fig. 2:**
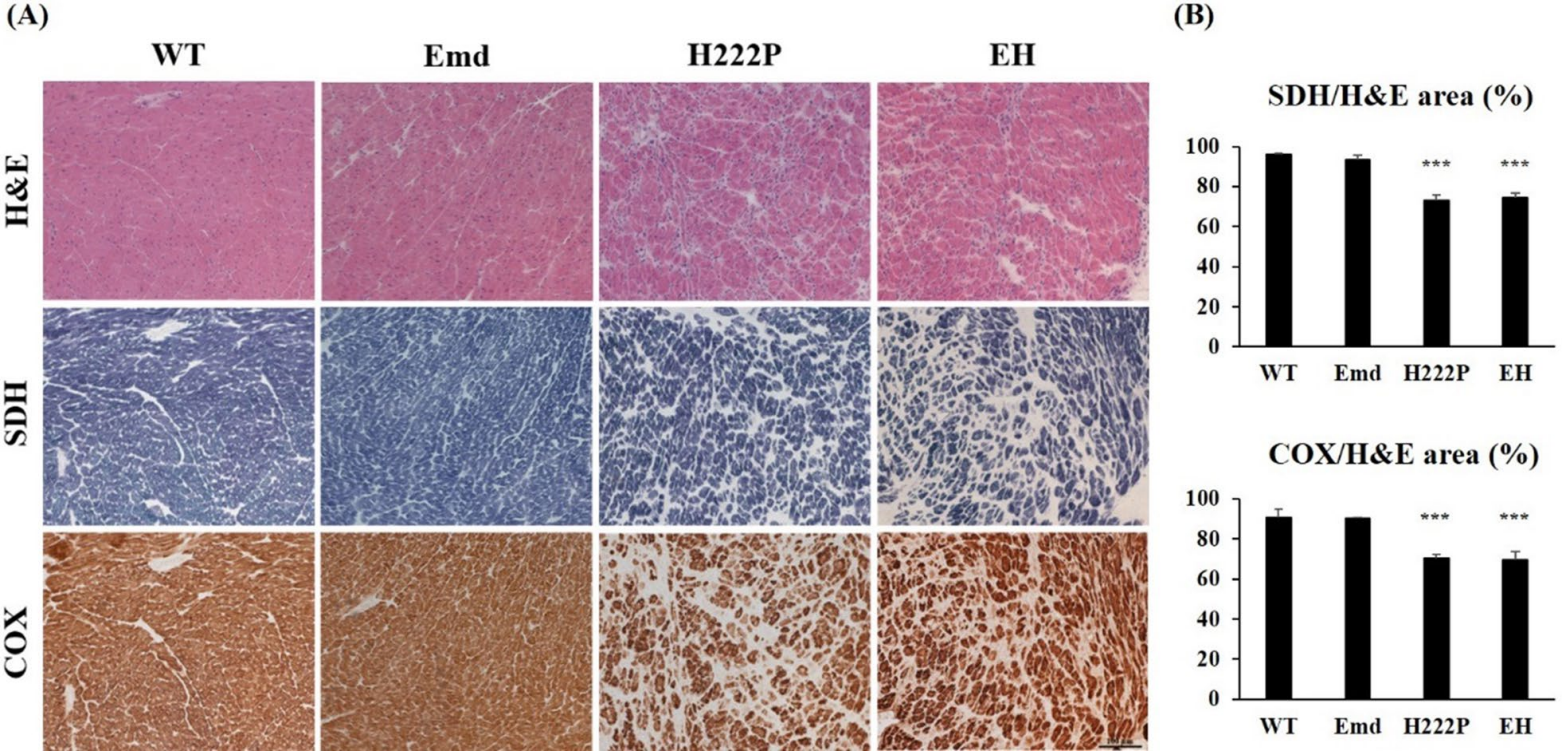
Histological images of cardiac muscle sections of WT and EDMD model mice. **A** H&E images showing an increase in the interstitial space between cardiomyocytes, and unevenly stained areas of SDH and COX in cardiac sections from H222P and EH mice at 30 weeks of age but not from Emd mice. Scale bar represents 100 µm. **B** Graphs represent the percentage of SDH-stained or COX-stained area normalized by the area of H&E stained cardiac muscle from serial sections (n = 3 for WT and Emd mice, and n = 4 for H222P and EH mice). ***P < 0.001 versus WT mice at 30 weeks of age

In our previous study, we found that EH mice had a reduced maximum speed of aerobic running capacity at 12 weeks of age, although their cardiac function remained normal [10]. In the present study, cardiac function and structure were evaluated by echocardiography and histological analysis in male WT and EDMD model mice at 18 and 30 weeks of age. Compared with WT mice, H222P and EH mice had significantly increased interventricular septum thickness at diastole, interventricular septum systolic thickness at systole, and left ventricular internal dimension at systole, and significantly decreased left ventricle posterior wall thickness at 18 and 30 weeks of age. Statistically significant decreased ejection fraction rates (%EFs) and fractional shortening rates (%FSs) were also observed in H222P and EH mice at these ages (Table 1). Emd mice had comparable results to WT mice in these cardiac parameters at the same ages. Cardiac dysfunction progressed similarly in H222P and EH mice that both %EFs and %FSs were significantly decreased in an age-dependent manner. The average of %EFs was reduced from 70.5 ± 6.2% at 18 weeks of age to 63.0 ± 5.8% at 30 weeks of age (*P* < 0.05) and that of %FSs was reduced from 33.9 ± 4.8% at 18 weeks of age to 28.4 ± 3.8% at 30 weeks of age (*P* < 0.05) in H222P mice. The average of %EFs was decreased from 72.0 ± 11.2% at 18 weeks of age to 58.1 ± 6.3% at 30 weeks of age (*P* < 0.01) and that of %FSs was reduced from 35.7 ± 7.9% at 18 weeks of age to 25.4 ± 3.9% at 30 weeks of age (*P* < 0.01) in EH mice. Histological observation of cardiac cryosections demonstrated an increase in interstitial fibrosis, and abnormal distribution of mitochondrial enzymes (SDH and COX) were indistinguishable in H222P and EH mice, but was not observed in Emd mice at 30 weeks of age (Fig. 2A). The intensity of SDH and COX stained area was significantly reduced both in H222P and EH mice at 30 weeks of age, compared with WT mice (Fig. 2B).

**Table 1:** Echocardiography data of male WT and EDMD model mice at 18 and 30 weeks of age

18 weeks of age	WT (*n* = 6)	Emd (*n* = 8)	H222P (*n* = 8)	EH (*n* = 8)
IVSd (mm)	0.93 ± 0.10	0.99 ± 0.16	0.66 ± 0.11 **	0.71 ± 0.17 *
LVIDd (mm)	3.50 ± 0.27	3.50 ± 0.30	3.77 ± 0.34	4.05 ± 0.40 *
LVPWd (mm)	0.73 ± 0.09	0.84 ± 0.20	0.58 ± 0.09	0.64 ± 0.10
IVSs (mm)	1.60 ± 0.14	1.73 ± 0.18	1.13 ± 0.12 ***	1.25 ± 0.19 ***
LVIDs (mm)	1.75 ± 0.24	1.85 ± 0.24	2.50 ± 0.39 **	2.64 ± 0.60 **
LVPWs (mm)	1.32 ± 0.12	1.34 ± 0.14	0.92 ± 0.13 ***	1.07 ± 0.16 *
EF (%)	86.8 ± 3.1	85.1 ± 3.3	70.5 ± 6.2 ***	72.0 ± 11.2 **
FS (%)	49.8 ± 4.1	47.4 ± 4.2	33.9 ± 4.8 ***	35.7 ± 7.9 ***
HR (BPM)	457 ± 17	483 ± 44	431 ± 31	458 ± 21

30 weeks of age	WT (*n* = 6)	Emd (*n* = 8)	H222P (*n* = 10)	EH (*n* = 12)
IVSd (mm)	0.98 ± 0.08	1.08 ± 0.12	0.57 ± 0.16 ***	0.76 ± 0.21 *^, #^
LVIDd (mm)	3.43 ± 0.14	3.68 ± 0.13	3.73 ± 0.27	3.73 ± 0.37
LVPWd (mm)	0.95 ± 0.12	0.83 ± 0.17	0.65 ± 0.07	0.84 ± 0.42
IVSs (mm)	1.78 ± 0.08	1.88 ± 0.15	1.10 ± 0.15 ***	1.15 ± 0.25 ***
LVIDs (mm)	1.68 ± 0.16	1.68 ± 0.18	2.69 ± 0.30 ***	2.79 ± 0.32 ***
LVPWs (mm)	1.63 ± 0.33	1.53 ± 0.17	0.92 ± 0.15 ***	1.13 ± 0.36 **
EF (%)	88.6 ± 2.6	90.2 ± 2.7	63.0 ± 5.8 ***	58.1 ± 6.3 ***
FS (%)	51.7 ± 3.8	54.2 ± 4.2	28.4 ± 3.8 ***	25.4 ± 3.9 ***
HR (BPM)	451 ± 55	448 ± 36	459 ± 52	445 ± 49

Electrocardiograms from long axis M-mode images were recorded, and cardiac morphology and function were evaluated by the levels of interventricular septum thickness at diastole (IVSd), left ventricular posterior wall thickness at diastole (LVPWd), left ventricular internal dimension at diastole (LVIDd), interventricular septum systolic thickness at systole (IVSs), left ventricular internal dimension at systole (LVIDs), left ventricular posterior wall thickness at systole (LVPWs), ejection fraction rate (EF), fractional shortening rate (FS), and heart rate (HR) beats per minute (BPM) during recording of mice at 18 and 30 weeks of age. *P < 0.05, **P < 0.01, ***P < 0.001 versus WT mice, and ^#^*P* < 0.05 versus H222P mice

### Myocardial fibrosis

**Fig. 3:**
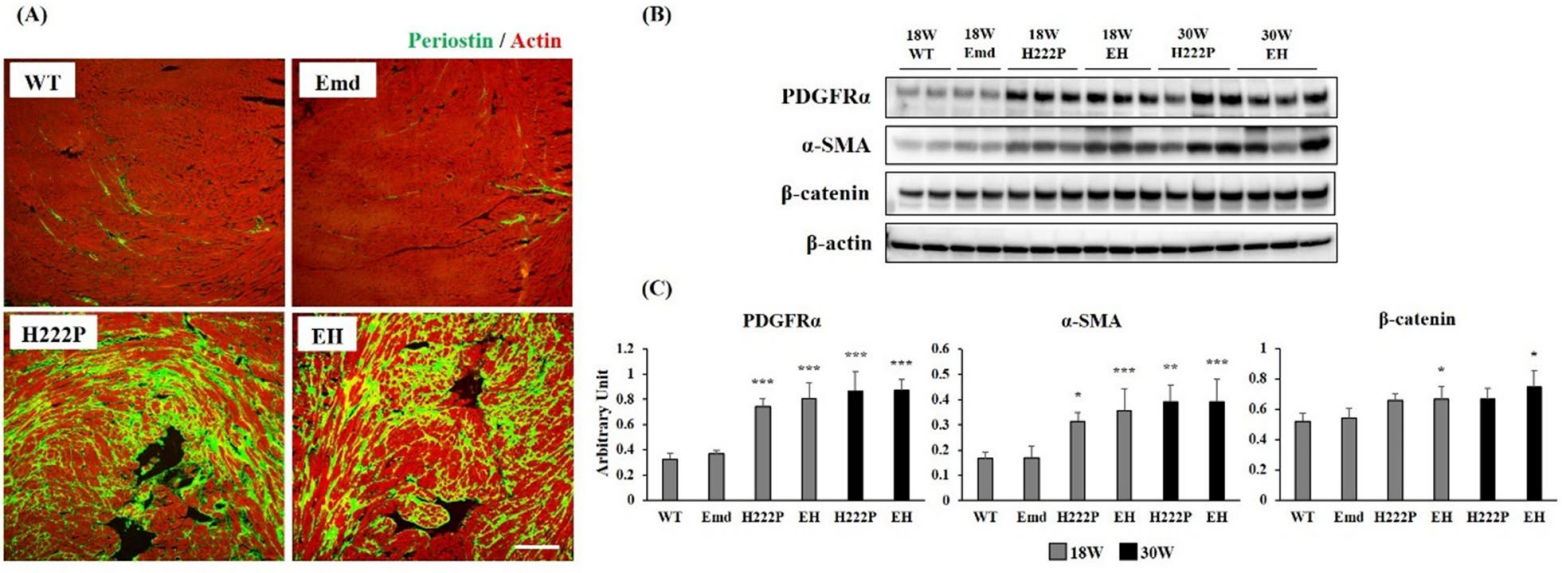
Evaluation of myocardial fibrosis by immunohistochemistry and western blotting. **A** Representative images of a section of the cardiac muscle from WT and EDMD model mice. Periostin-positive fibrotic areas were prominent around cardiomyocytes in the left ventricles compared with those in the right ventricles of both H222P and EH mice at 30 weeks of age. Scale bar represents 100 µm. **B** Western blots were performed on four individual samples from WT and Emd mice at 18 weeks of age, and six individual samples from H222P and EH mice at 18 and 30 weeks of age. **C** Graphs represent the quantification of fibrotic markers PDGFRα, α-SMA, and β-catenin levels normalized to the level of β-actin. *P < 0.05, **P < 0.01, and ***P < 0.001 versus WT mice at 18 weeks of age

In cardiomyopathy, cardiac muscle atrophy or reduction results in increased interstitial space, which is likely compensated by fibrotic tissues. Immunostaining of cardiac sections indicated that interstitial fibrosis is prominent around cardiomyocytes in the left ventricles of H222P and EH mice at 18 weeks of age (Fig. 3A). An increase in the protein expression levels of the fibrosis mediators PDGFRα, α-SMA, and β-catenin were detected in the hearts of H222P and EH mice at 18 weeks of age, and these protein levels were remained increased at 30 weeks of age (Fig. 3B and C). No appreciable difference in cardiac fibrosis was observed between H222P and EH mice, and also between WT and Emd mice at both 18 and 30 weeks of age.

### Mitochondrial content

The abnormal distribution of mitochondria in cardiomyocytes is known to be a hallmark of the cardiomyopathy in H222P mice [11]. In this study, altered mitochondrial enzyme activities were suggested in both H222P and EH mice from SDH and COX staining of cardiac sections (Fig. 2A and B). We predicted that the expression of major mitochondria regulators is also reduced in EDMD model mice. However, western blot analysis demonstrated that protein levels of PGC1α (a master regulator of mitochondrial biogenesis), NDUFAF1 (a protein involved in complex I assembly), ATP5A1 (a mitochondrial ATP synthase), and TFAM (the most abundant protein associated with mitochondrial DNA) were comparable among cardiac samples from WT and the three EDMD mouse models (Additional file 1: Fig. S1). There were no gradual changes in the expression levels of the above proteins in H222P and EH mice from 18 to 30 weeks of age.

### Signaling mediators related to cardiomyopathy

**Fig. 4:**
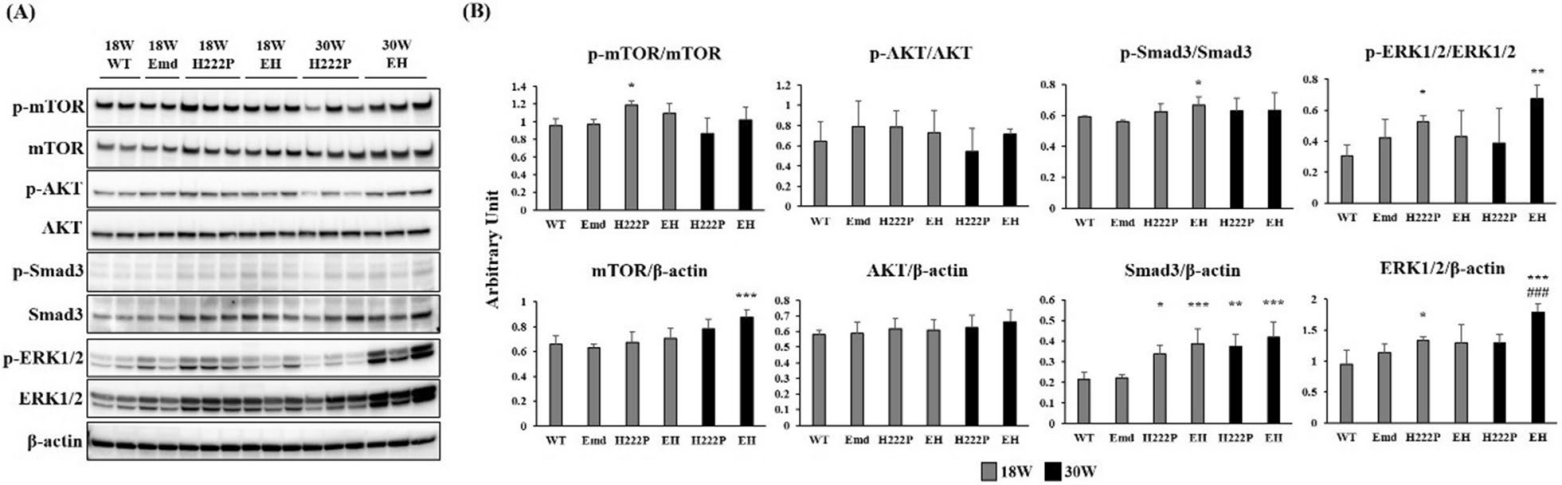
Western blot analysis of signaling mediators affected by cardiomyopathy in EDMD mice.** A** Immunoblot images of signaling mediators affected by cardiomyopathy are shown. **B** Graphs represent the ratio between the phosphorylated forms of mTOR, AKT, Smad3, and ERK1/2, normalized to the total level of each target protein. Total levels of these proteins were normalized to the level of β-actin (*n* = 4 for WT and Emd at 18 weeks of age, and *n* = 6 for H222P and EH mice at 18 and 30 weeks of age). *P < 0.05, **P < 0.01, and ***P < 0.001 versus WT mice at 18 weeks of age. ^###^*P* < 0.001 versus H222P mice at 30 weeks of age

**Fig. 5:**
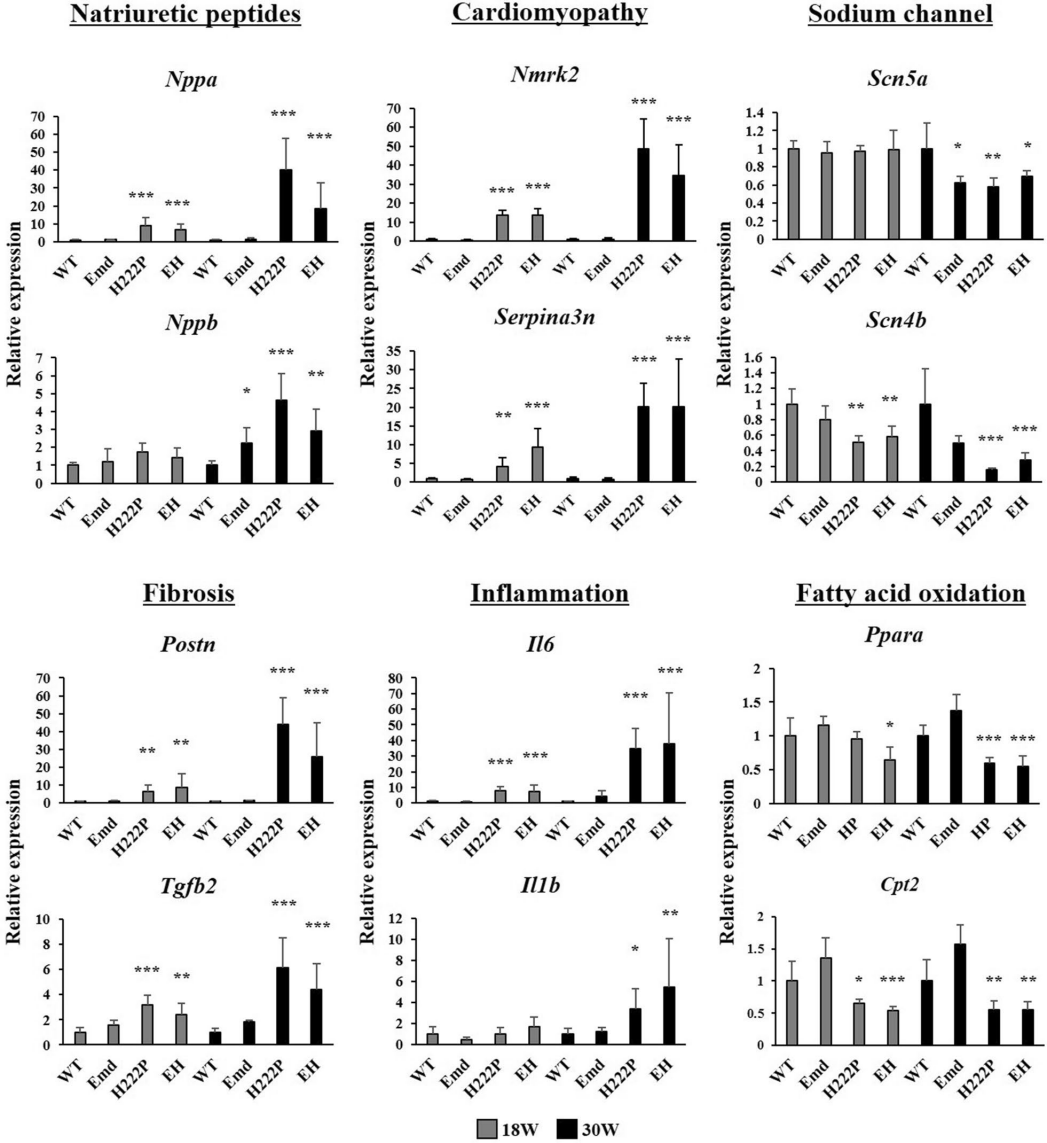
Quantification of genes associated with cardiomyopathy by RT-qPCR. Relative mRNA levels of *Nppa*, *Nppb*, *Nmrk2*, *Serpina3n*, *Scn5a*, *Scn4b*, *Postn*, *Tgfb2*, *Il6*, *Il1b, Ppara and Cpt2* were normalized by *Gapdh* and shown as the fold increase to WT mice. *P < 0.05, **P < 0.01, and ***P < 0.001 versus age-matched WT mice

Previous studies using H222P mice demonstrated that several signaling mediators are involved in the development of cardiomyopathy [9, 12, 13]. Among the detectable markers of cardiomyopathy, we evaluated the activation of mTOR, AKT, Smad3, and ERK1/2 to investigate the association of emerin and lamin A/C in the regulation of signaling pathways associated with cardiomyopathy (Fig. 4A and B). Reduced cardiac function in H222P mice has been reported to be associated with increased mTOR signaling and impaired autophagic flux [14]. Compared with WT mice, phosphorylated mTOR was significantly increased in H222P mice at 18 weeks but not at 30 weeks of age, and total mTOR level was significantly increased in EH mice at 30 weeks, whereas phosphorylated AKT and total AKT levels were comparable among the EDMD mice of different ages. Increased fibrosis in cardiac muscles is partially driven by cytokines, such as TGF-β. The upregulation of *Tgfb2* mRNA expression was observed both in H222P and EH mice at 18 and 30 weeks of age (Fig. 5). TGF-β signaling induces the phosphorylation of Smad, which subsequently activates ERK1/2 signaling to aggravate cardiac fibrosis [15]. We found increased levels of total Smad3 and ERK1/2 protein levels in both H222P and EH mice, whereas phosphorylated Smad3 and ERK1/2 protein levels were variable among H222P and EH mice. The phosphorylation of these proteins were identically detected in WT and Emd mice at 18 and 30 weeks of age (data not shown). Taken together, the deficiency of emerin did not induce a more severe cardiac phenotype in EH mice at these ages even though total ERK1/2 protein level was significantly upregulated in EH mice at 30 weeks of age.

### The expression of cardiomyopathy-associated genes

Several genes have been identified to be involved in the development of cardiomyopathy caused by *Lmna* mutations [11]. In addition to the levels of *Nppa* and *Nppb*, which encode natriuretic peptide A and B, respectively, *Nmrk2*, encoding a nicotinamide riboside kinase that is involved in NAD + biosynthesis was significantly upregulated in the cardiac muscle of H222P and EH mice. Similarly, the serine proteinase inhibitor A3 gene *Serpina3n*, a biomarker of heart failure, was identically increased in H222P and EH mice, compared with WT mice. These genes were significantly increased in both H222P and EH mice in an age-dependent manner. Downregulation of the arrythmia-associated sodium voltage-gated channel genes *Scn5a* and *Scn4b* was also prominent in EDMD model mice, including Emd mice at 30 weeks of age. In addition, increased levels of genes associated with fibrosis (*Postn* and *Tgfb2*) and inflammation (*Il6* and *Il1b*) in H222P and EH mice were confirmed. Representative genes associated with fatty acid oxidation (*Ppara* and *Cpt2*) in cardiac muscle were significantly downregulated in H222P and EH mice. Taken together, several cardiomyopathy-associated genes were altered in both H222P and EH mice, compared with WT and Emd mice: however, were not notably changed by emerin deficiency in mice with the *Lmna* mutation (Fig. 5).

## Discussion

In this study, we assessed the progression of cardiac abnormalities in EDMD murine models, to elucidate the effects of emerin deficiency together with the *Lmna*^H222P/H222P^ mutation. EDMD is an inherited disorder that predominantly affects striated muscles. Cardiac abnormalities occur after skeletal muscle dysfunctions, and are usually prominent after the second to third decade of life [16]. Cardiac symptoms are progressive, and are the most severe aspect in EDMD patients. EDMD2 caused by mutations in *LMNA* leads to more severe ventricular dysfunction and heart failure compared with emerin-deficient EDMD1 [17]. Emd mice show no muscular dystrophy, and only a mild atrioventricular conduction delay after 40 weeks of age [8], and die around 18 months of age (data not shown). H222P mice die by 9 months of age owing to dilated cardiomyopathy; however, only mild skeletal muscle changes have occurred when cardiac dysfunction is prominent [9]. We previously reported that EH mice demonstrate early onset severe skeletal muscle dystrophy at 12 weeks of age. At the same age, EH mice were found to have a normal growth curve, and no cardiac abnormalities were observed, similar to H222P mice [10]. In the present study, we analyzed whether the deficiency in emerin coupled with the *Lmna*^H222P/H222P^ mutation exacerbates cardiac function at 18 and 30 weeks of age and shortens the life span of mice.

Both male and female EH mice had a similar average life span to H222P mice. In the original report of H222P mice, female H222P and EH mice were found to tend to live longer than male mice [9]. We used male mice for the further analysis of cardiac function, and kept the female mice for production of the next generation. We demonstrated in a previous study that male H222P and EH mice at 12 weeks of age have normal cardiac functions, and show no signs of cardiac abnormalities compared to WT mice [10]. Consistent with previous reports [18–20], H222P mice in the present study showed cardiac symptoms at about 18 weeks of age, which then worsened in an age-dependent manner. EH mice had similar trends in cardiac parameter changes on echocardiography to H222P mice. In particular, the decrease in %EF and %FS of H222P and EH mice compared with WT mice were prominent both at 18 and 30 weeks of age. Moreover, the substantial increase in interstitial fibrosis was similarly detected in H222P and EH mice.

The mechanisms underlying the cardiac abnormalities of H222P mice have been studied in detail previously, and several markers have been identified as a target for drug treatment [21–25]. In particular, ERK1/2 was reported to be upregulated in the cardiac muscle of emerin-null mice [26], and this upregulation was more pronounced in H222P mice [12]. Total protein levels of ERK1/2, which may be a target signaling pathway for treatment, were significantly increased in EH mice compared with H222P mice at 30 weeks of age, whereas the ratio of phosphorylated to total protein was not constantly increased. This result indicates that emerin deficiency in H222P mice could lead to the additional increase in ERK signaling in cardiac muscle. Further studies are needed to elucidate whether this modulation is a cardiac muscle-specific change or not.

Interestingly, genes associated with cardiomyopathy in H222P mice were similarly altered in EH mice. We did not detect any significant differences in the expression of those genes between H222P and EH mice. A possible reason for this variability is the effects of the different genetic backgrounds of H222P mice [27]. H222P mice, together with the Emd and EH mice used in this study have a C57BL/6 J background. Previous reports analyzing signaling mediators used the original H222P mice with the 129S2 strain background. Vignier et al. demonstrated that the genetic background of mice substantially affects the severity of cardiac involvement of the *Lmna* mutation [27]. Therefore, variations in the levels of signaling mediators may occur owing to the effects of different genetic backgrounds.

The direct cause of death in H222P and EH mice is still unsolved. Our results indicate the possible mechanism in which cardiac necrosis and fibrosis are progressed, and decreased ejection fraction rate represents cardiac failure. Moreover, decrease in genes associated with sodium channel leads to arrhythmia, and alternations in genes related to fatty acid oxidation would represent a chronic energy crisis due to the progression of cardiomyopathy.

All the findings from the comparison of striated muscles of H222P and EH mice demonstrated that emerin plays distinctive roles in skeletal and cardiac muscles. EH mice have exacerbated skeletal muscle phenotypes but not cardiac abnormalities compared with H222P mice. Emerin, a lamin A-interacting protein, is highly conserved and ubiquitously expressed in all differentiated cells. Emerin deficiency is known to alter signaling pathways associated with myogenesis in skeletal muscle samples from EDMD1 patients [28] and Emd mice [29, 30]. Moreover, recent studies indicated that emerin is an important nuclear membrane protein for nuclear polarity in myoblasts [31], and for nuclear migration to reseal injured muscle membranes [32]. Yet, Emd mice are essentially normal, including their skeletal muscles, and no adverse phenotypes in other organs have been reported. The expression of compensating nuclear membrane proteins is thought to be a factor that attenuates the dystrophic phenotypes in emerin-null mice [29, 33]. Further studies are needed to elucidate the tissue-specific roles of nuclear membrane proteins, including emerin and lamin A/C.

In conclusion, we demonstrated that genetic suppression of emerin does not worsen cardiac dysfunction in H222P mice. Although H222P mice recapitulate several important features of the cardiac phenotypes observed in patients with laminopathies, H222P mice have a limited lifespan before Emd mice exhibit mild conduction disorder around 50 weeks of age. This study has the inherent limitations associated with the use of *Lmna*^H222P/H222P^ mice to observe emerin deficiency in cardiac muscle; however, EH mice demonstrate a relatively similar progression of cardiomyopathy to H222P mice. Unlikely, skeletal muscle abnormalities are prominent in EH mice compared with H222P mice; therefore, EH mice are suitable murine models for elucidating the mechanism of muscular dystrophy in EDMD patients.

### Supplementary Information

**Additional file 1: Table S1.** Primer sequences used for real-time PCR analysis. **Figure S1.** Western blot analysis of mitochondrial proteins from cardiac muscles in WT and EDMD mice. **A** Immunoblot images of mitochondrial markers are shown. **B** Graphs represent the quantification of PGC1α, NDUFAF1, ATP5A1, and TFAM levels normalized to the level of β-actin.

## Data Availability

The original contributions presented in the study are included in the article. Further inquiries can be directed to the corresponding author.
