# Supplementary material for: Emerin deficiency does not exacerbate cardiomyopathy in a murine model of Emery–Dreifuss muscular dystrophy caused by an LMNA gene mutation
Source: J Physiol Sci. 2023 Nov 8;73:27. doi: 10.1186/s12576-023-00886-0 (PMC10717240; doi:10.1186/s12576-023-00886-0)

**Additional file**

**Additional file Table. Primer sequences used for real-time PCR analysis**

| **Gene** | **Forward primer** | **Reverse primer** |
| --- | --- | --- |
| *Nmrk2* | 5′-AAACTCATCATAGGCATTGGAGG-3′ | 5′-GTCCTGGGGCTTGAAGAAGT-3′ |
| *Serpina3n* | 5′-GCCTCGTCAGGCCAAAAAG-3′ | 5′-TGAACGTGTCAAGAGGGTCAA-3′ |
| *Nppa* | 5′-CAGAATCGACTGCCTTTTCC-3′ | 5′-GGGGGTAGGATTGACAGGAT-3′ |
| *Nppb* | 5′-ACCCAGGCAGAGTCAGAAAC-3′ | 5′-ACAAGATAGACCGGATCGGA-3′ |
| *Scn5a* | 5′-TGCTGAATAAGGGCAAAACCA-3′ | 5′-GCTGAAGAGCGAATGTACCAAAA-3′ |
| *Scn4b* | 5′-GGAACCGAGGCAATACTCAGG-3′ | 5′-CCGTTAATAGCGTAGATGGTGGT-3′ |
| *Postn* | 5′-TCACCGTTTCGCCTTCTTTA-3′ | 5′-CACCTTCAAAGAAATCCCCA-3′ |
| *Tgfb2* | 5′-TTGTTGAGACATCAAAGCGG-3′ | 5′-ATAAAATCGACATGCCGTCC-3′ |
| *Il6* | 5′-ACCAGAGGAAATTTTCAATAGGC-3′ | 5′-TGATGCACTTGCAGAAAACA-3′ |
| *Il1b* | 5′-GGTCAAAGGTTTGGAAGCAG-3′ | 5′-TGTGAAATGCCACCTTTTGA-3′ |
| *Ppara* | 5′-AGTCAAGGTGTGGCCCAAGGT-3′ | 5′-GTCTATCGGACACTAGCGGAGGC-3′ |
| *Cpt2* | 5′-CAGCACAGCATCGTACCCA-3′ | 5′-TCCCAATGCCGTTCTCAAAAT-3′ |
| *Gapdh* | 5′-TTGATGGCAACAATCTCCAC-3′ | 5′-CGTCCCGTAGACAAAATGGT-3′ |

**Additional file Figure**


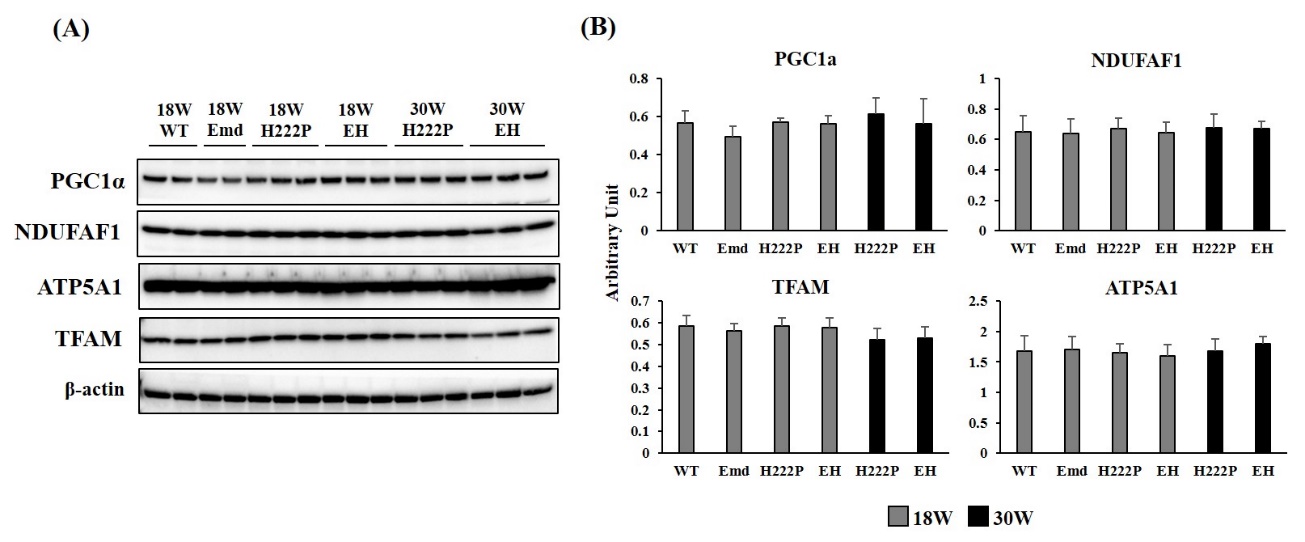

Supplement: Supplementary file 1 — Additional file 1: Table S1. Primer sequences used for real-time PCR analysis. Figure S1. Western blot analysis of mitochondrial proteins from cardiac muscles in WT and EDMD mice. A Immunoblot images of mitochondrial markers are shown. B Graphs represent the quantification of PGC1α, NDUFAF1, ATP5A1, and TFAM levels normalized to the level of β-actin. [file 12576_2023_886_MOESM1_ESM.docx]
